# An open-label clinical trial of oral transmucosal haloperidol and oral transmucosal olanzapine in the treatment of terminal delirium at home

**DOI:** 10.1186/s13063-022-06238-4

**Published:** 2022-04-14

**Authors:** Xiao-Juan Lyu, Adrian David Kan, Poh-Heng Chong, Keegan Lin, Yung-Hua Koh, Zhi-Zheng Yeo

**Affiliations:** 1 HCA Hospice, Singapore, Singapore; 2 IPOS International, Singapore, Singapore

**Keywords:** Haloperidol, Olanzapine, Terminal delirium, Home hospice, Palliative care, End of life

## Abstract

**Background:**

The phenomenon of restlessness, agitation, or cognitive disturbances experienced by dying patients is well-known in palliative care; more than half of these patients will experience delirium symptoms at end-of-life. When not identified early and effectively managed, delirium symptoms could lead to caregiver and patient distress and harm.

**Methods:**

Eighty patients with a prognosis of 7 days or less will be recruited for an open-label randomised control trial. The two arms compare oral-transmucosal haloperidol 2.5 mg vs olanzapine 5 mg over 72 h. The severity of agitation, delirium and toxicities of treatments will be compared at the 24th, 48th and 72nd hour after drug administration.

**Discussion:**

This trial is the first to compare anti-psychotics in the management of delirium at the dying stage in the home hospice setting using the oral transmucosal route. Ethical considerations, as well as recruitment procedures, are discussed.

**Trial registration:**

This study was registered in ClinicalTrials.gov – identifier NCT04750395

## Administrative information

Note: the numbers in curly brackets in this protocol refer to SPIRIT checklist item numbers. The order of the items has been modified to group similar items (see http://www.equator-network.org/reporting-guidelines/spirit-2013-statement-defining-standard-protocol-items-for-clinical-trials/).

**Table:** 

Title {1}	Protocol of an open-label clinical trial of oral transmucosal haloperidol and olanzapine in the treatment of terminal delirium
Trial registration {2a and 2b}.	ClinicalTrials.gov Identifier - NCT04750395
Protocol version {3}	Version 1; 19 May 2021
Funding {4}	Pending review – Community Care Research Grant by Agency for Integrated Care Singapore
Author details {5a}	*Xiao-Juan Lyu*^*1*^*; Adrian David Kan*^*1*^*; Poh-Heng Chong*^*1*^*; Keegan Lin*^*1*^*; Yung-Hua Koh*^*2*^*; Zhi-Zheng Yeo*^*1*^ ^*1*^*HCA Hospice Care* ^*2*^*IPOS International*
Name and contact information for the trial sponsor {5b}	HCA Hospice Care
Role of sponsor {5c}	HCA Hospice Care is the site of recruitment of participants. Staff are supported by the sponsor to carry out the study. Ultimate authority over the report will belong to the investigators.

## Introduction

### Background and rationale {6a}

Terminal delirium is a common syndrome in palliative care. It occurs within the final days of life in between 25 and 88% of dying patients and is perceived to be one of the most difficult end-of-life symptoms to manage [1]. In contrast to other types of delirium that are treatable (and hence reversible), terminal delirium is a complex and refractory syndrome that is often multi-factorial in aetiology. It is characterised by a diminished level of consciousness, an inability to focus or maintain attention, and a disturbance of cognition and perception (e.g. hallucination, agitation, and restlessness).

Delirium could have a profound impact on the well-being of the patients as well as the family caregivers. Family caregivers perceived a high level of stress both physically and psychologically in the management of delirious patients [2]. Similar distress was also observed by the investigators in the home-based hospice setting. Within community care, pharmacological therapies are utilised to manage the syndrome, the most commonly used being neuroleptics haloperidol and olanzapine.

Haloperidol is the first of the butyrophenone series of major antipsychotics. The mechanism of action of haloperidol for the treatment of psychotic disorders is unclear. However, its efficacy could be mediated through its activity as an antagonist of central dopamine type 2 receptors. Haloperidol also binds to alpha-1

adrenergic receptors, but with lower affinity, and displays minimal binding to muscarinic, cholinergic, and histaminergic (H_1_) receptors.

Olanzapine is a second-generation thienobenzodiazepine antipsychotic which displays potent antagonism of serotonin 5-HT2A and 5-HT2C, dopamine D1-4, histamine H1, and alpha1-adrenergic receptors. Olanzapine shows moderate antagonism of 5-HT3 and muscarinic M1-5 receptors, and weak binding to GABA-A, BZD, and beta-adrenergic receptors. Although the precise mechanism of action in schizophrenia and bipolar disorder is not known, the efficacy of olanzapine is thought to be mediated through combined antagonism of dopamine and serotonin type 2 receptor sites.

A recent Cochrane systematic review evaluating the effectiveness and safety of drug therapies to manage delirium symptoms in terminally ill adults has only found four clinical studies that included haloperidol and olanzapine, with high risks of bias and low to very low quality of evidence. Due to the absence of evidence from robust clinical trials, current recommendations are based on consensus or expert opinion, and clinical experience in the care of patients at the end of life [3]. Given these considerations, we believe there is immense value in comparing the therapeutic effects of haloperidol versus olanzapine within the hospice home care setting at the end of life; other researchers have encouraged this investigation to be pursued [4].

Hence, we propose an open-label randomised clinical trial to compare the effects of haloperidol and olanzapine in the management of terminal delirium in home hospice patients who are imminently dying. Key outcome measures are the reduction of delirium symptoms and the reduction of agitation. Secondary outcome is the adverse effect burden on patients.

### Objectives {7}

This study is a non-inferiority trial that aims to compare the effect of Oral Transmucosal (OT) haloperidol and olanzapine in the management of terminal delirium in dying patients receiving home hospice care. The study’s secondary objective is to record the frequency and severity of toxicity from treatment. It is hypothesised that OT Haloperidol will reduce the severity of agitation and of terminal delirium as effectively as OT olanzapine, i.e. Haloperidol will not perform “unacceptably worse” than olanzapine.

### Trial design {8}

A prospective, parallel, open-label, randomised controlled trial was designed. Informed consent will be obtained from the patient’s proxy at home. After obtaining consent, a primary caregiver at home (who may or may not be the proxy) will be identified to administer the trial medication and make observations as specified in the trial procedures. When a home hospice clinician identifies a patient, who meets the inclusion criteria, his or her proxy will be approached by the study team and invited to participate in the study. Patients will be randomly assigned to receive either OT haloperidol or OT olanzapine as treatment.

After commencement of the first dose of medication, each patient will be observed by their family caregiver and the attending clinician over 72 h (less if the patient dies earlier). Time-points for data collection will be at 24 h, 48 h, and 72 h after the first commencement of medication. Table 1 outlines the trial in the form of the SPIRIT figure.

**Table 1:**
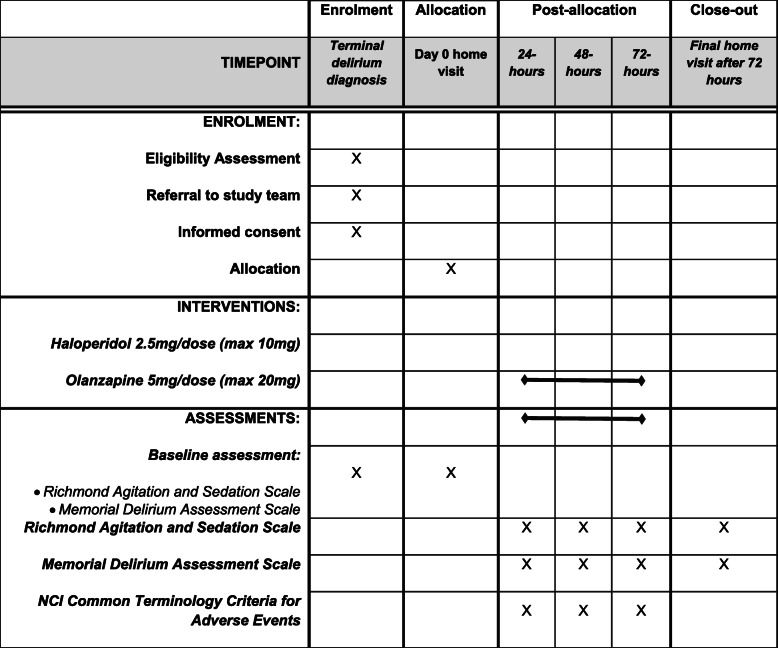
SPIRIT figure for clinical trial

This study defines “terminal delirium” as an episode of delirium that occurs during the dying phase, usually 72 h before death [5]. Episodes of delirium in the dying phase may be described as “terminal restlessness” or “terminal delirium”. The use of the label “terminal” implies a causal relationship between the dying phase and the delirium, although the aetiology is often multi-factorial. Given that the trajectory of death is often uncertain, patients who survive up to 7 days after their commencement in the trial will be included.

## Methods: participants, interventions, and outcomes

### Study setting {9}

The study will be conducted within HCA Hospice Care (HCA). HCA is the largest home hospice provider in Singapore, looking after more than 60% of patients living in the community with life-limiting conditions. The average length of service is three months. Patient census is around 900–1000 on any given day, supported by five teams of multi-disciplinary professionals across the island, including on-site 24/7 support.

### Eligibility criteria {10}

The Inclusion criteria for the trial are as follows:

1. Patient is above 21 years of age.
2. Patient was diagnosed with a terminal illness and is receiving end-of-life care at home.
3. Patient is assessed to be acutely dying (estimated prognosis of 3 days or less).
4. Patient is diagnosed by the primary palliative care provider to have delirium, as described in the DSM-V [6]

The Exclusion criteria are as follows:

1. Patient does not have a caregiver at home.
2. Patient has a prior history of dementia, psychosis, schizophrenia or any other mental health issue followed up by psychiatrists or other specialists.
3. Patient is currently receiving or has been administered haloperidol or olanzapine less than a week before participating in the study.
4. Patient has known allergies or adverse reactions to haloperidol or olanzapine.

### Who will take informed consent? {26a}

This study will be conducted within one site in the country of Singapore. As this study will recruit patients who are experiencing terminal delirium, the patients would fulfil the definition of lacking mental capacity under Section 4 of the Mental Capacity Act, “a person who lacks capacity is one who lacks mental capacity in relation to a matter, if at the material time, this person is unable to make a decision for own self in relation to the matter because of an impairment of, or a disturbance in the functioning of, the mind of brain.” [7]

For this study, a proxy (or person responsible) for the patient would be approached to provide consent on behalf of the patient. Proxy consent was suggested as a suitable method for consent collection, given the patient’s absent or fluctuating capacity to consent. It was successfully used in dementia and delirium research and was reported to have been acceptable to patients and their caregivers [8]. In most situations, the proxy will also be the primary caregiver, who will administer the medication dosages per instructions, monitor the patient, and ensure that the patient’s care needs are met. However, there are exceptions in which the proxy and caregiver are separate individuals. In this case, the identified caregiver will be included during consent-taking and receive the participant information sheet.

Section 7 of the Human Biomedical Research Act [9] states that, where there is a need to recruit adults who lack mental capacity, appropriate consent for the adult must be obtained via a valid proxy, either:

(i) From a donee or deputy (if existing or in force, to be verified in ways feasible by the research team at the time) who is authorised to give consent to the biomedical research on behalf of the adult, or
(ii) In the case where there is no pre-designated donee or deputy, consent is obtained from any of the following persons, in the following order of priority:
  - Spouse
  - Adult child
  - Parent or Guardian
  - Adult sibling
  - Any other person named by the adult as someone to be consulted on the matter in question or on matters of that kind.

The proxy for the patient will be approached by a study team member who does not provide direct medical or hospice care to the patient. This is to reduce the risk of compulsion through an established patient-provider relationship. The member will provide more detailed information about the study to the proxy. If agreeable, the proxy will sign the informed consent form and the patient will be recruited into the study. The proxy can refuse to participate, with no compromise to the patient’s care; the patient’s primary palliative care team will continue to provide medical care and hospice support in the usual way outside of a research study. The study team will assure the proxy of this fact before informed consent is collected.

### Additional consent provisions for collection and use of participant data and biological specimens {26b}

Not applicable.

## Interventions

### Explanation for the choice of comparators {6b}

Haloperidol and olanzapine are two antipsychotic drugs commonly used by palliative care clinicians. Haloperidol is an older drug that has been used historically, while olanzapine is a newer option, purportedly for its lower side effect profile [3]. One recent study compared haloperidol and olanzapine injections in 100 hospitalized patients and found that they were similar in efficacy, as well as safety [10].

The dosage of haloperidol varied across previous studies on terminal delirium management with a general range of 1.5-15 mg per day. However, lower dose of haloperidol regime showed no significant benefit in reducing the severity of delirium symptom; a study by Li n[11] found that the loading doses were 5 mg and titrated to 10 mg per day with a maximum dose of 15 mg/day. This has led to a significant improvement of delirium rating scale across time periods. In our study design, the doses of OT haloperidol for the assigned group of patients are 5–10 mg per day based on the symptom burden.

Current published evidence for the dose of olanzapine in managing terminal delirium is scarce although it has been increasingly used for general delirium treatment with a recommended dose of 2.5–30 mg per day. The equivalent dose for the first- and second-generation antipsychotics based on 100 mg chlorpromazine are 1.5–2 mg haloperidol to 4–5 mg olanzapine [12]. In our study, the doses of OT olanzapine for the assigned group of patients are 10–20 mg per day.

Other than injections, both medications can be administered in oral transmucosal forms. This route of administration is an important option for patients in the community care setting; in many situations, the caregiver would be responsible for administrating medication. However, they may not be trained to provide injections or may feel that injections are distressing. OT administration serves as a more viable, less stressful and safer alternative.

No evidence is available for comparing efficacy between these two drugs in this route of administration. This serves as the impetus for the study.

### Intervention description {11a}

Every 24 h, patients will receive two regular doses of either OT haloperidol 2.5 mg or OT olanzapine 5.0 mg with an interval of 12 h. Patients may also receive up to two breakthrough doses within a minimum interval of 1 h from the last dose when needed. In total, up to four doses of medication will be prepared. The dosage schedule is provided below in Table 2.

**Table 2:** Dosage schedule for clinical trial

	Timing	Haloperidol	Olanzapine
First 24 h	First dose	2.5 mg	5.0 mg
	12 h later	2.5 mg	5.0 mg
	First breakthrough	2.5 mg	5.0 mg
	Second breakthrough	2.5 mg	5.0 mg
Second 24 h	First dose	2.5 mg	5.0 mg
	12 h later	2.5 mg	5.0 mg
	First breakthrough	2.5 mg	5.0 mg
	Second breakthrough	2.5 mg	5.0 mg
Third 24 h	First dose	2.5 mg	5.0 mg
	12 h later	2.5 mg	5.0 mg
	First breakthrough	2.5 mg	5.0 mg
	Second breakthrough	2.5 mg	5.0 mg
	Total dose prepared	30.0 mg (10.0 mg/day)	60.0 mg (20.0 mg/day)

The haloperidol preparation is a solution with concentration of 2 mg/mL of haloperidol. The medication is administered via dropper, where 20 drops is equal to 1 mL (1 drop = 0.05 mL). Standardized doses of 2.5 mg of haloperidol (25 drops or 1.25 mL) of the haloperidol preparation would be administered for each dose.

Olanzapine preparation is a 5 mg oro-dispersible tablet. Standardized doses of 5 mg will be delivered each time a dose is required.

After obtaining consent, the patient will be randomized into either the haloperidol or olanzapine group. Instructions on how to administer the medication will be provided to the caregivers; caregivers will also be advised to serve breakthrough doses of the assigned anti-psychotic drug as needed. Basic demographic information, including the patient’s age, sex, primary diagnosis, and co-morbidities, will be collected.

All relevant trial materials are prepared in packs with the following materials:

- The required amount of medication (haloperidol or olanzapine),
- Rescue medication—subcutaneous midazolam with syringe and needle,
- A copy of the patient information sheet and informed consent form,
- Instructions for the caregivers on how to administer medication, and
- A copy of the case report form.

The investigators will provide the packs to the caregivers of enrolled patients. Additionally, the investigators will conduct daily clinical reviews at home over 72 h. They will confirm that the dosages used and the dosages documented are congruent.

Subcutaneous injectable midazolam would be prepared as a rescue medication in the event delirium symptoms remain uncontrolled and distressing after the trial medication is depleted. Rescue medications will be used in any one of the following situations:

1. Within any of the 24-h periods, the patient remains uncontrollably agitated, despite all prepared doses of the treatment having been applied.
2. Patient is unable to tolerate OT medication.
3. Patient experiences severe toxicities from trial medication.

The caregiver will receive detailed instructions for the trial included with the case report forms. The forms will also provide support resources such as the hospice hotline and investigators’ contact number. Per clinical practice, the caregiver will give the first dose of medication under the supervision of the study team. Additionally, they will be instructed on how to rate the patient’s agitation and will provide the baseline rating with the investigator’s support. The study team physician will assess the patient’s baseline symptom severity.

Alongside drug therapy, usual non-pharmacological interventions will be provided to all patients. These interventions include (i) regularly orientating the patient; (ii) keeping the room bright during the day; (iii) minimizing the use of tubes, catheters, physical restraints, or other immobilizing devices; and (iv) minimizing unnecessary disturbances to the patient.

### Criteria for discontinuing or modifying allocated interventions {11b}

The study will be terminated for that patient in any one of the following situations:

1. Midazolam injectable rescue medication is used, or the allocated daily dosage limit is reached.
2. Toxicities related to the trial medication were observed to be intolerable.
3. Proxy asks to withdraw the patient from the study.
4. Patient is admitted to the hospital.

The study may be terminated at any point during the trial. In that event, the investigators will record the reason for cessation, and make a final assessment of the patient, where applicable. The patient will continue to receive support from their palliative care providers. Data from the patient will be included for analysis up to the point of discontinuation; subsequent time points will not include that patient.

The proxy or the patient may choose to withdraw participation from the study at any time, without compromising further treatment using other drugs or alternative dosage regimen. The patient’s primary palliative nurse will be informed, and they will continue to arrange and provide medical care and hospice support for the patient, in the usual way outside of research. They will advise how persisting symptoms should be managed, outside of the study.

If voluntary withdrawal occurs, the patient will be asked to continue scheduled evaluations, complete an end of study evaluation, and be given appropriate care under medical supervision until the symptoms of any adverse event resolve or the patient’s condition becomes stable.

### Strategies to improve adherence to interventions {11c}

Instructions for how to administer medication will be provided to the caregivers by the investigators. Moreover, the patients and their caregivers have access to a 24/7 on-call service, which they can call to make clarifications and to receive medical support when needed.

At each time-point (i.e. daily), the investigators will conduct a home visit to ensure that the medication has been appropriately administered and that the ratings have been recorded.

### Relevant concomitant care permitted or prohibited during the trial {11d}

There are no limitations on other medications, herbs, vitamins, and mineral supplements other than study agents while participating in the study. Additionally, if there were any assistance required after-hours (related to the trial or not), the caregivers will have access to the emergency helpline. The study team will also be notified to render assistance if needed. All medications (prescription and over the counter), vitamin and mineral supplements, and/or herbs taken by the participant will be documented and used to control for confounding factors if needed.

### Provisions for post-trial care {30}

As this trial involves patients whose prognoses are between 3 and 7 days, patient death is expected by the end of the trial. However, in the event the patient outlives the prognoses and survives, the patient’s primary nurse and staff will continue to provide standard palliative care to the patient. At this point, medical advice and end-of-life decisions will be made independently from the study. The patient’s survival (whether alive, discharged, or deceased) will be monitored up to day 7 of recruitment. If the patient is still alive after day 7, their data will be removed from the main analysis as they would no longer fall under the inclusion criteria of an imminently dying patient.

### Outcomes {12}

The primary outcome is the reduction in delirium severity as measured with the Memorial Delirium Assessment Scale (MDAS). Secondary outcomes are the reduction in the severity of agitation as measured with the Richmond Agitation-Sedation Scale modified for palliative care (RASS), and the frequency and severity of medication toxicities, as measured using the National Cancer Institute Common Terminology Criteria for Adverse Events (NCI-CTCAE). These data will be collected over four time-points: (i) baseline, (ii) 24 h after commencement, (iii) 48 h after commencement, and (iv) 72 h after commencement.

### Participant timeline {13}

**Fig. 1:**
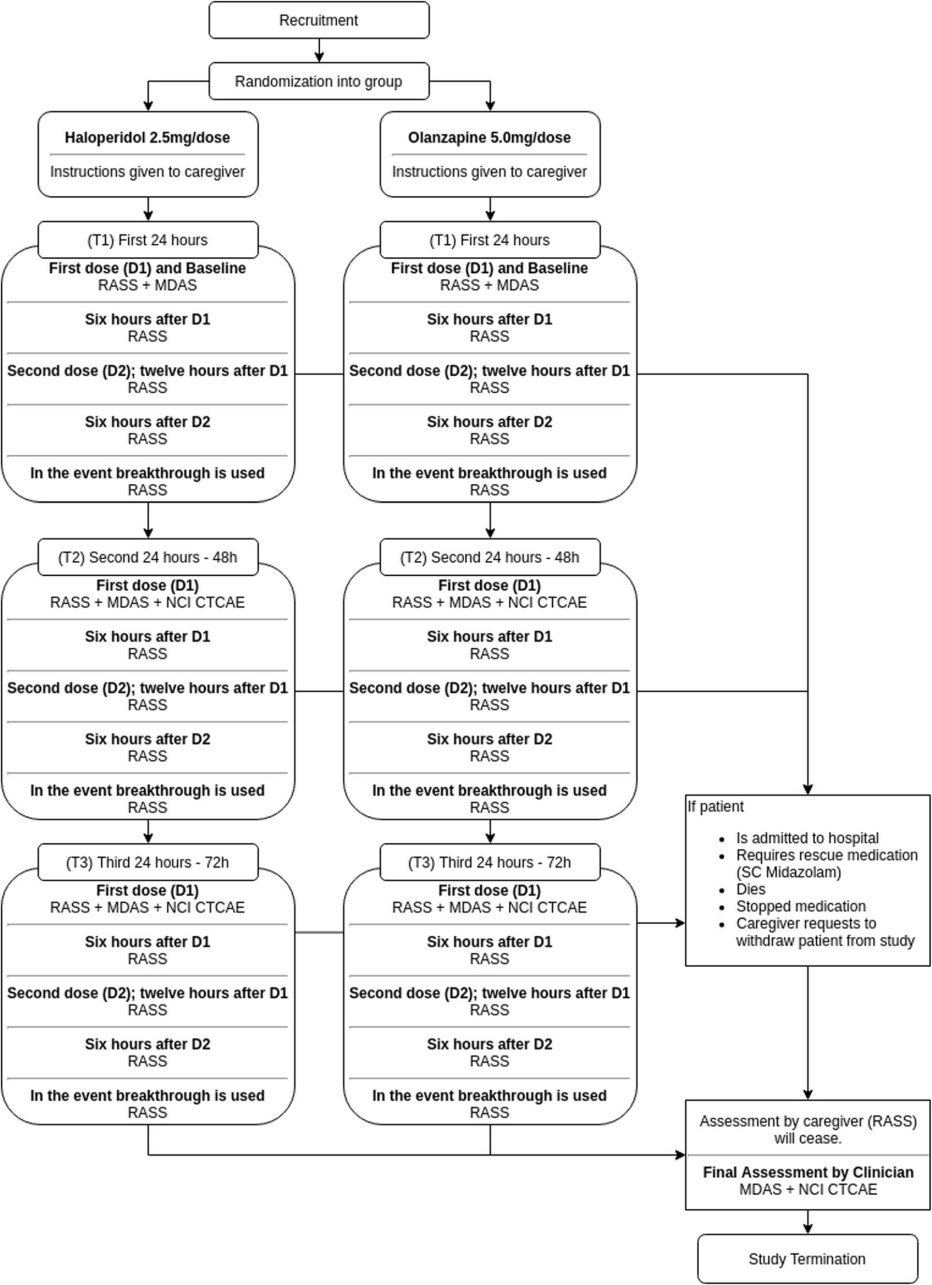
Flowchart of clinical trial procedures

The participant timeline is shown in Fig. 1.

### Sample size {14}

Based on validation studies by Breitbart et al. [13] and Lawlor et al. [14], the mean scores from the Memorial Delirium Assessment Scale for patients with delirium is roughly 18 (SD = 7.64). Jain et al. [10] reported that using either haloperidol or olanzapine led to a nearly 55% reduction in patients’ MDAS score (7–8 points). Based on this, we estimate 2 points to be the minimal important difference between haloperidol and olanzapine; a 2-point difference between the treatment arms is equivalent to a 25% difference in the reduction of a patient’s MDAS score. Table 3 details sample size calculations, using the parameters as presented above.

**Table 3:** Sample size calculation for repeated measures design

Sample size calculation for repeated measures design	
Standard deviation (drawn from literature review)	7.64
Estimated minimal important difference (effect size)	2
Target power (1-β error probability)	.80
Target *alpha*	.05
Estimated required size per treatment arm	32
Estimated minimum sample size for two arms	64
Target recruitment size (accounting for 30% attrition)	80

### Recruitment {15}

HCA’s clinical staff attending to the patients under its service will assist in recruitment. As they continue to provide standard palliative care to the patient, the clinical staff will:

(i) Identify patients who meet inclusion criteria and share preliminary information with caregivers,
(ii) Confirm the diagnosis of delirium, and
(iii) Advise caregivers on appropriate non-pharmacological management.

The clinical staff of the hospice service will be briefed about the study and will refer patients to the research team. The research team will explain the details of the study and collect informed consent from them.

## Assignment of interventions: allocation

### Sequence generation {16a}

The randomisation sequence is generated before the start of the study with 1:1 allocation using random block sizes of 2 and 4. With each successive recruitment, patients will be assigned to the treatment arm consecutively based on their sequence on the generated list. The patient will receive a Trial Identification Number based on their position of the sequence.

To begin the trial, Study personnel will collect assigned trial medication from the organization’s pharmacy and bring along to the home visit. The material packs prepared for the trial will then be marked in the order of the randomised allocation; this allows for the investigators to more easily and more quickly identify the pack to pick up and deliver to the patient.

### Concealment mechanism {16b}

Not applicable.

### Implementation {16c}

The study team’s research executive will generate the randomisation sequence and number the prepared medication packs accordingly. These packs will be placed in secure storage within the organization’s pharmacy. The investigators, when visiting the patient to collect informed consent, will subsequently collect the packs and deliver them to the family.

## Assignment of interventions: blinding

### Who will be blinded {17a}

This study was designed as an open-label study and thus there was no blinding between trial participants, care providers or the researchers.

### Procedure for unblinding if needed {17b}

Not applicable.

## Data collection and management

### Plans for assessment and collection of outcomes {18a}

The properties of the MDAS, RASS, and NCI-CTCAE are described as follows:

#### The Memorial Delirium Assessment Scale

MDAS is a ten-item, four-point clinician-rated scale designed to quantify the severity of delirium in medically ill patients [13]. It has very good psychometric properties, with high reliability (*r* = .91) and good discriminant and concurrent validity. Though the scale was intended to assess patients based on all ten items, it was suggested that items in MDAS can be pro-rated in the event the patient is not able to communicate (Lawlor, et al., 2000). To conduct assessments as well as to ensure patient’s safety, the study team physician will make regular home visits to assess the patient at the time of the first dose, and then at 24 h, 48 h, and 72 h after the first dose.

#### The Richmond Agitation-Sedation Scale modified for palliative care

The RASS is a simple observational instrument assessing levels of sedation and agitation. It requires no patient input and ranges from + 4 (overly combative) to − 5 (unarousable). It is considered less time-consuming and easier to use than other similar instruments [15]. Developed for adult intensive care unit patients, the scale demonstrated strong inter-rater reliability in that setting. A modified version was designed for use in the palliative care setting, which produced acceptable psychometric properties. Hui, et al. had caregivers using the RASS to assess patients, which gave ratings similar to clinicians [16]. As part of scoring calibration with the caregivers, the attending study team members will guide the caregivers to provide the first score during the time of the first dose. Subsequently, caregivers will chart the patient’s agitation using the RASS every 6 h after the first dose. If breakthrough medication is required, the caregiver will also chart the time of administration and provide a RASS score.

#### National Cancer Institute Common Terminology Criteria for Adverse Events [17]

Possible side effects of taking either drug include over sedation and extrapyramidal symptoms (akathisia, extrapyramidal disorder, and spasticity). To track these adverse events and their severity, the study team physician will rate observed adverse events using the scale provided by the criteria at 24 h, 48 h and 72 h.

The trial will be conducted for up to 72 h after recruitment; the trial may be stopped earlier in the event of the patient’s death or to ensure the patient’s safety and well-being. If the patient completes the trial and survives, the patient will continue to receive support from their palliative care providers. Patients who survive beyond 7 days after recruitment will be excluded from per-protocol analysis, as they no longer fit the inclusion criteria for the study, i.e. no longer imminently dying.

### Plans to promote participant retention and complete follow-up {18b}

Due to the nature of terminal delirium, patients’ prognoses are short, and they may become deceased before completing all time points in the study. As such, daily visits and assessments by the investigators will be conducted. This serves to ensure compliance with the intervention, completeness of data collection, as well as allow the clinicians to render support for the family where needed.

In the event the patient dies before the final assessment, the investigators will still make a final home visit to make a final assessment with the caregivers, and to collect the case report form and remaining medication. The family will continue to receive psychosocial support from their palliative care provider, including subsequent bereavement support.

### Data management {19}

Each trial will have an associated case report form, which will be reviewed by the visiting investigator after each visit to ensure the accuracy of recorded data.

Data will initially be recorded on hardcopies of the case report forms by both the clinician and caregiver before they are transcribed to a Microsoft Excel file. The file is web-based and access-secured; only the investigators are able to access the file via their hospice organizational account. For electronic data, they will be stored in an encrypted and password-protected excel file. At the completion of data collection and initiation of analysis, participant identifiers will be removed from the file. The de-identified file will remain while the file with participant identifiers will be destroyed

For hardcopy data, they will be stored in designated locked cabinet(s) or room(s) that are accessible to authorized study personnel only. Only the patient’s Trial Identification Number will be used to identify the hardcopy.

Records for all participants, including case report forms, all source documentation (containing evidence to study eligibility, history and physical findings, laboratory data, results of consultations, etc.) as well as Institutional Review Board (IRB) records and other regulatory documentation should be retained by the PI in a secure storage facility. The records should be accessible for inspection and copying by authorised authorities.

### Confidentiality {27}

The electronic file will contain only de-identified data and will be stored in a secure folder in the hospice intranet which is only accessible to research staff. Hardcopy data will be stored up to 2 years after the publication of the study. At the end of two years, the hardcopy will be shredded and disposed of in accordance with MOH’s guidelines for personal and sensitive information.

The patient’s Hospice Identification number (known as the HAH number) will be collected for the purposes of extracting demographic information from the electronic medical records. Demographic data, including age, sex, and primary diagnoses, will be extracted for descriptive presentation.

### Plans for collection, laboratory evaluation and storage of biological specimens for genetic or molecular analysis in this trial/future use {33}

Not applicable.

## Statistical methods

### Statistical methods for primary and secondary outcomes {20a}

For the first primary outcome, changes to the severity of delirium over three 24-h periods, as measured with the MDAS, would be analysed using repeated-measures, between-factors ANOVA. If a statistically significant difference is found between medications, post hoc analysis will be conducted to analyse the change in MDAS scores at each time-point.

The second outcome, changes in the patient’s agitation, will be measured using the RASS score. Similarly, the repeated-measures, between-factors ANOVA would be used to analyse the differences in scores.

Additional outcomes of interest are possible toxicity effects due to the medication, which is measured using NCI CTCAE. For each group, the frequency of toxicities (Akathisia, Extrapyramidal disorder, and spasticity) for each group will be compared.

### Interim analyses {21b}

Not applicable.

### Methods for additional analyses (e.g. subgroup analyses) {20b}

Not applicable.

### Methods in analysis to handle protocol non-adherence and any statistical methods to handle missing data {20c}

Given the unstable state of the patient, and the community setting being a more difficult-to-control environment, protocol non-adherence may occur due to a variety of real-world factors. The investigators will document instances of non-adherence and assess if the patient would need to be withdrawn from the trial; patient safety and comfort would be prioritised.

To handle protocol non-adherence, analysis will be done on an intention-to-treat analysis basis; participants would be classified according to the treatment they were originally randomised. This analysis method would best reflect the original trial design.

### Plans to give access to the full protocol, participant-level data, and statistical code {31c}

Only the study investigators will have access to the raw data and hardcopies. De-identified and aggregated data may be obtained from the research team upon reasonable request.

The sponsor institution (HCA) will permit study-related monitoring, audits and/or IRB review and regulatory inspections, providing direct access to source data/document.

## Oversight and monitoring

### Composition of the coordinating centre and trial steering committee {5d}

This clinical trial is supported by HCA’s research department. A Research Executive coordinates organisational support for the trial and manages the research team’s meeting and supports the research team’s overseeing of the trial.

### Composition of the data monitoring committee, its role and reporting structure {21a}

A data monitoring committee (DMC) was not sought for this clinical trial due to the following reasons. First, the trail is open label. Second, the medications used are widely used in both hospital and community, with a well-known risk profile that are minimal and manageable by clinical staff and the caregivers.

### Adverse event reporting and harms {22}

Related adverse events are defined as events where there is a reasonable possibility that the adverse event (AE) may have been caused by participation in the clinical trial. Relatedness may be graded as one of three options: definitely, probably, or possibly.

Any complaints, adverse event, serious adverse event, or incidence must be reported to the Principal Investigator. Follow-up information will be actively sought and submitted as it becomes available. All related AEs will be recorded and filed as a Research Incident Report Form in the patient’s study file.

For all AEs, the study team and the medical director of HCA will be notified to review the incident and discuss changes or improvements to be made to address the complaint. For serious AEs, a report will be submitted to both the IRB and the Health Sciences Authority (HSA).

### Frequency and plans for auditing trial conduct {23}

Audits from trial conduct will be done monthly by the study team. An audit will include a review of subject eligibility, informed consent process, protocol compliance, disease outcome, adverse events and toxicity, data quality, regulatory documentation, and investigational product records.

### Plans for communicating important protocol amendments to relevant parties (e.g. trial participants, ethical committees) {25}

Only the study investigators would be allowed to propose modifications to the trial protocols. In the event that a modification of the protocols must be enacted, trials and recruitment will be stopped. Any modifications to the protocols planned must first be submitted to the IRB for review and approval, followed by a submission to HSA. Once approved, the protocol may be modified per the proposal and the participants shall be notified.

### Dissemination plans {31a}

Findings from this clinical trial should be analysed and published in a peer-reviewed journal at the end of the study. The study will credit all investigators listed in this protocol as authors. Acknowledgement may be given to any members who have contributed to the conduct of the trials and the writing of the report.

## Discussion

To compare the efficacy of two pharmacological treatments of terminal delirium in dying patients on hospice care, a prospective, randomised, open-label trial will be conducted at home setting. The two groups of patients will be receiving OT haloperidol and olanzapine respectively.

Findings from this study will contribute to scarce evidence of anti-psychotics/ neuroleptics use in the treatment of terminal delirium for patients receiving home hospice care. It would inform future practice on appropriate medication to treat delirium during the dying phase. With optimised care, the end-of-life experience of patients and their caregivers will be improved.

Some facts were discussed during the study design. First, a blinded design will not be possible or necessary. Due to limited clinical evidence of pharmacological treatment of terminal delirium in the home environment, the additional blinding procedures are hazardous and not providing any added value. Second, proxy consent has been chosen instead of advance consent. To approach patients before the onset of terminal delirium is neither feasible nor appropriate considering the potential unnecessary stress caused to the vulnerable group of patients in their terminal phase.

## Trial status

The study was reviewed and approved by the Agency for Integrated Care Institutional Review Board (AIC-IRB). Clinical Trial Authorisation is pending from the Health Sciences Authority Singapore (HSA). Recruitment is projected to start in August 2021.

## Abbreviations

OT: Oral transmucosal
DSM-V: Diagnostic and Statistical Manual of Mental Disorders – Fifth Edition
IRB: Institutional Review Board
HSA: Health Sciences Authority
MDAS: The Memorial Delirium Assessment Scale
RASS-PAL: The Richmond Agitation-Sedation Scale modified for palliative care
NCI CTCAE: National Cancer Institute Common Terminology Criteria for Adverse Events
SC: Subcutaneous
ANOVA: Analysis of variance
